# Supplementary material for: The causal relationship between the human gut microbiota and pyogenic arthritis: a Mendelian randomization study
Source: Front Cell Infect Microbiol. 2024 Nov 26;14:1452480. doi: 10.3389/fcimb.2024.1452480 (PMC11629706; doi:10.3389/fcimb.2024.1452480)
Supplement: Supplementary file 1 [file DataSheet1.zip › mendelian test/ebi-a-GCST90027570.csv_leave_one_out.pdf]

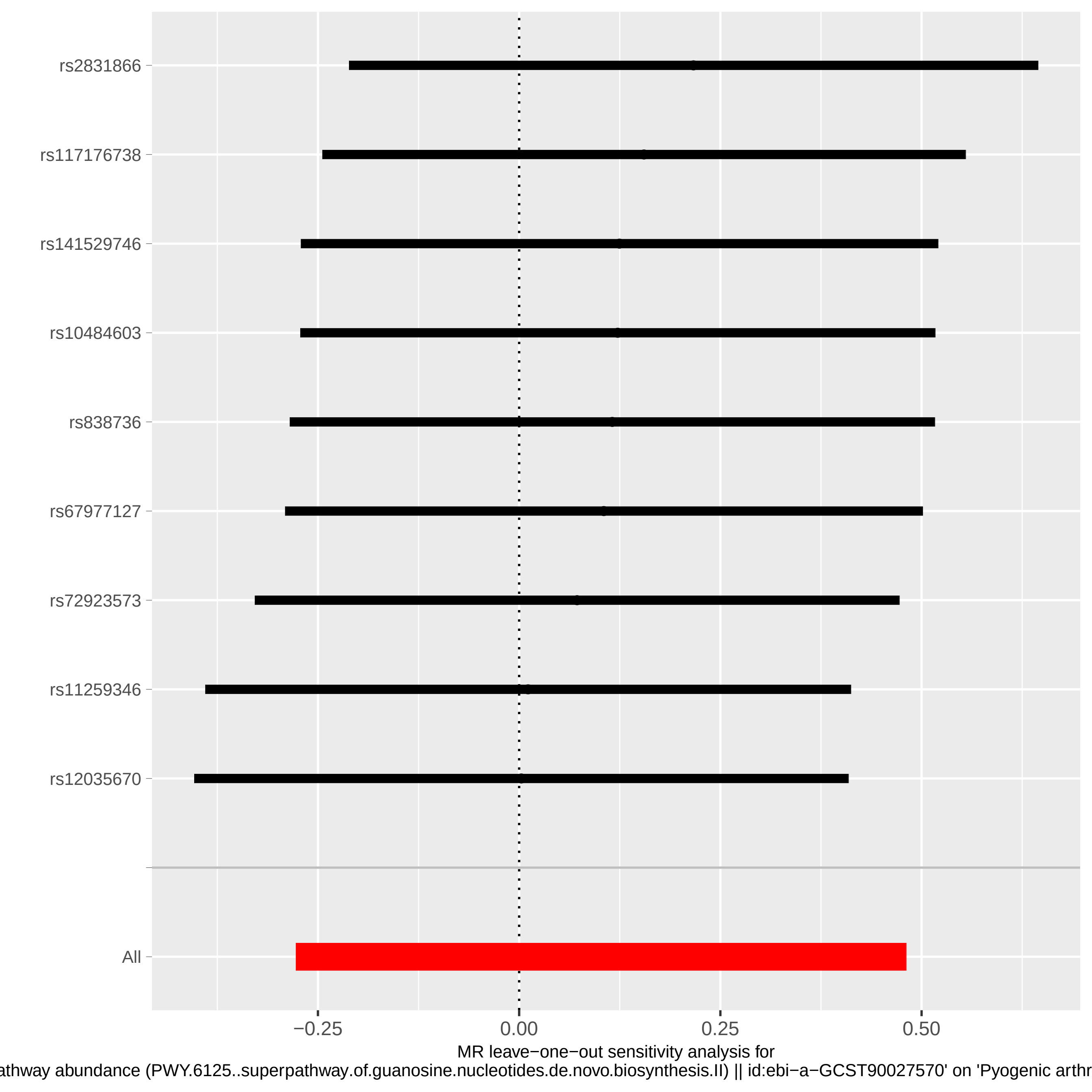
rs2831866
rs117176738
rs141529746
rs10484603
rs838736
rs67977127
rs72923573
rs11259346
rs12035670
All
−0.25
0.00
0.25
0.50
MR leave−one−out sensitivity analysis for
athway abundance (PWY.6125..superpathway.of.guanosine.nucleotides.de.novo.biosynthesis.II) || id:ebi−a−GCST90027570' on 'Pyogenic arthr
